# Impact of Microbiota on Irritable Bowel Syndrome Pathogenesis and Management: A Narrative Review

**DOI:** 10.3390/medicina61010109

**Published:** 2025-01-13

**Authors:** Mhd Bashir Almonajjed, Mahdi Wardeh, Abdallah Atlagh, Abdulrahman Ismaiel, Stefan-Lucian Popa, Flaviu Rusu, Dan L. Dumitrascu

**Affiliations:** 1 Faculty of Medicine, “Iuliu Hatieganu” University of Medicine and Pharmacy, 400006 Cluj-Napoca, Romania; m.basheralmounajed@gmail.com (M.B.A.); mahdi.wardeh2003@gmail.com (M.W.); atlagha1@yahoo.fr (A.A.); 2 2nd Department of Internal Medicine, “Iuliu Hatieganu” University of Medicine and Pharmacy, 400006 Cluj-Napoca, Romania; popa.stefan@umfcluj.ro (S.-L.P.); flaviurusu@yahoo.com (F.R.); ddumitrascu@umfcluj.ro (D.L.D.)

**Keywords:** irritable bowel syndrome, microbiota, gut–brain axis, disorders of gut brain interaction (DGBI)

## Abstract

Irritable bowel syndrome (IBS) is a prevalent gastrointestinal disorder, affecting 3–5% of the global population and significantly impacting patients’ quality of life and healthcare resources. Alongside physical symptoms such as abdominal pain and altered bowel habits, many individuals experience psychological comorbidities, including anxiety and depression. Recent research has highlighted the critical role of the gut microbiota in IBS, with dysbiosis, characterized by an imbalance in microbial diversity, frequently observed in patients. The gut–brain axis, a bidirectional communication network between the gut and central nervous system, plays a central role in the development of IBS symptoms. Although interventions such as probiotics, prebiotics, synbiotics, and fecal microbiota transplantation (FMT) have demonstrated potential in modulating the gut microbiota and alleviating symptoms, their efficacy remains an area of ongoing investigation. This review examines the interactions between the gut microbiota, immune system, and brain, emphasizing the need for personalized therapeutic strategies. Future research should aim to identify reliable microbiota-based biomarkers for IBS and refine microbiome-targeted therapies to enhance patient outcomes.

## 1. Introduction

Irritable bowel syndrome (IBS) affects 3–5% of the world’s population and is diagnosed using the Rome IV criteria, which are symptom-based [1]. While the impact on mortality is unknown, IBS has a major influence on quality of life, especially through linked psychiatric illnesses such as anxiety and depression, resulting in higher healthcare usage and decreased productivity.

Research emphasizes the significance of gut microbiota in IBS, with dysbiosis, characterized by reduced microbial diversity, reported in many patients. Specific bacterial families, such as *Firmicutes* and *Proteobacteria*, are implicated, and a decrease in butyrate-producing bacteria may contribute to symptom onset by compromising intestinal barrier integrity [2]. Probiotics have shown promise in treating IBS symptoms, highlighting the microbiome’s role in the condition [2].

The brain–gut connection, which has a long history, has been scientifically validated using modern imaging techniques. According to research, gut stimuli can trigger brain regions involved in emotion regulation, and gastrointestinal dysfunction frequently precedes neurological diseases such as Parkinson’s disease [3]. This emphasizes the significance of gut health in general neurological and emotional well-being, underlining the need to better understand gut–brain connections in IBS patients.

The global prevalence of IBS varies due to factors such as food, ethnicity, and healthcare systems. IBS-D (diarrhea-predominant) and IBS-C (constipation-predominant) account for around 30% of cases, with women having a greater incidence [4]. The disorder has a substantial impact on daily living, limiting productivity and social participation. Some patients are willing to give up years of life for symptom alleviation. Recent research has connected altered gut microbiota to IBS, implying that bile acids, psychosocial variables, and genetic predispositions all contribute to IBS pathogenesis [4]. However, the particular microbiome signature associated with IBS severity and treatment response is still being investigated.

The pathogenesis of IBS is complicated, involving elements such as visceral hypersensitivity and gut microbiota changes, with the gut–brain axis playing a key role in symptom development. While studies on probiotics such as *Lactobacillus* and *Bifidobacterium* spp. demonstrated promise, their clinical importance is unknown [5]. IBS patients typically have altered gut microbiota, including decreased *Bifidobacterium* and increased *Bacteroides*, but the cause and stability of these alterations are still being explored [5]. The lack of specific biomarkers interferes with the diagnosis and the management, while genetic predisposition and psychological variables also contribute to the disease progression. The Rome IV criteria also indicate a continuum in gastrointestinal diseases, with symptom overlap common, confounding diagnosis and comprehension of disorders of gut–brain interaction (DGBI) [6].

While the significance of probiotics in IBS treatment is debated, synbiotics have shown promise in relieving symptoms, particularly in IBS-D patients [6]. Furthermore, fecal microbiota transplantation (FMT) is emerging as a potential therapy option [6]; however, its efficacy and safety must be further investigated.

This review explores the function of gut microbiota in the development and treatment of IBS. It investigates differences in gut microbiota composition among IBS subtypes, the gut–brain axis, and their roles in symptom development. Furthermore, it emphasizes the roles of biofilms and small intestinal bacterial overgrowth (SIBO) in IBS pathogenesis. Current microbiome-targeted therapeutics, including probiotics, prebiotics, synbiotics, and fecal microbiota transplantation (FMT), are evaluated alongside dietary interventions to determine their impact on gut microbiota and symptom alleviation. The emphasis is on personalized therapy techniques, with a focus on identifying research gaps and providing future approaches for improving microbiome-based diagnostics and therapeutics in IBS.

## 2. The Gut Microbiome in Health and Disease

The gut microbiota is critical to human health since it ferments dietary fibers, produces short-chain fatty acids (SCFAs), and regulates the immune system [6]. A healthy and diversified microbiome is essential for intestinal health and general well-being. Healthy individuals often have a diverse microbial makeup, characterized by beneficial bacteria such as *Lactobacilli* and *Bifidobacteria* [7]. The gut microbiota is predominantly composed of four phyla: *Firmicutes*, *Bacteroidetes*, *Actinobacteria*, and *Proteobacteria*, all of which play important roles in metabolic processes and immunological function [8].

The gut microbiota develops early in childhood and is impacted by a variety of factors, including nutrition and antibiotic exposure, which can alter microbial populations [8]. Individual microbial makeup varies significantly, influenced by eating habits, age, and lifestyle choices. According to research, microbiome alterations might cause imbalances, which contribute to gastrointestinal problems [6]. In healthy individuals, the microbiota makeup is dominated by beneficial species that aid in gastrointestinal homeostasis [9].

Perinatal variables, such as method of delivery and maternal education, have a major impact on the development of IBS, with a strong link to cesarean delivery. These factors have an impact on early life microbial profiles, which may increase IBS risk later in life [10].

## 3. Alterations in Gut Microbiota in IBS Patients

Alterations in gut microbiota have also been associated to immunological activation and intestinal barrier dysfunction, particularly in post-infectious IBS (PI-IBS), where previous infections can cause chronic symptoms [11]. Evidence indicates that molecular mimicry between microbial antigens and host proteins may contribute to chronic inflammation and nerve damage [11]. A systematic review found a relationship between gastroenteritis and IBS, and the overall prevalence of PI-IBS was 14.5% [12]. Compared to bacterial and viral enteritis, protozoal infections pose a greater risk for the development of PI-IBS [11]. This is linked to the stimulation of inflammatory processes, exposure to exogenous substances, and increased intestinal permeability [11]. Furthermore, gut dysbiosis, which includes reductions in good bacteria like *Bifidobacterium* and *Lactobacillus* and increases in dangerous species like *Enterobacteriaceae*, has been linked to IBS [11]. However, the variability of available studies makes it difficult to identify a consistent microbial signature for IBS, underlining the importance of subtype-specific research and microbiota-targeted therapy.

Functional gastrointestinal disorders (FGIDs) are common but poorly understood due to the absence of obvious organic abnormalities, complicating diagnosis and therapy. The Rome IV criteria redefined FGIDs as DGBI, emphasizing the importance of psychological comorbidities including anxiety and depression [13]. This review also emphasizes the gut–brain axis and the microbiome’s critical roles in DGBI, which includes IBS and functional dyspepsia [13]. New research suggests that the gut microbiota regulates gut motility, visceral sensitivity, and even brain activity, with neurological, immunological, and metabolic pathways supporting bidirectional communication [13]. Microbial metabolites that affect the gut–brain axis, such as serotonin, tryptophan, tryptamine, and SCFAs, modify these effects. Stress-induced dysbiosis and comorbid illnesses are among the psychiatric diseases linked to gut microbiota composition [11]. Given that each person’s microbiome is distinct, individualized treatment approaches, particularly diet-based therapies, are recommended to address individual variability.

Numerous phyla that make up the gut microbiota are essential to preserving gut homeostasis. *Enterococcus*, *Ruminococcus*, *Clostridium*, *Lactobacillus*, *Faecalibacterium*, *Roseburia*, and *Eubacterium* are among the *Firmicutes* that are involved in the metabolism of amino acids, carbohydrates, and lipids as well as the transformation of bile acids and the creation of cholesterol [2,8]. Along with aiding in the synthesis of vitamins K2, B1, B2, B6, B7, B9, and B12, they also support the integrity of the intestinal epithelial barrier and immunological response, which guards against enteric infections [2,8]. *Bacteroidetes*, which include taxa like *Bacteroides* and *Prevotella*, have related roles in immunological modulation, metabolic pathways, and appetite regulation [8,11]. Vitamin production (K2, B1, B2, B6, B7, B9, and B12), bile acid metabolism, and protection against enteric infections are all aided by *Actinobacteria*, which are represented by *Bifidobacterium* and *Corynebacterium* [2,8]. Finally, *Proteobacteria*, which include *Shigella*, *Escherichia*, and *Desulfovibrio*, are important in the metabolism of amino acids and can affect gut disease when their populations become dysregulated [8,11].

**Figure 1:**
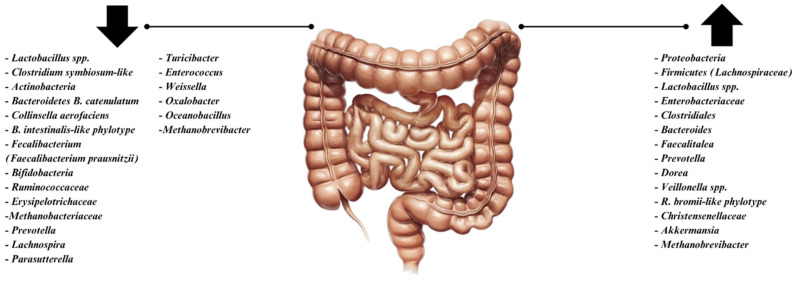
Microbiota alterations in IBS. (The figure is adapted with modifications from Surdea-Blaga et al., 2024, Microbiome in irritable bowel syndrome: advances in the field—A scoping review [25]).

In contrast to healthy microbiota, IBS patients have dysbiosis, which is characterized by diminished microbial diversity and microbial population imbalance. (Figure 1 demonstrates the alterations that can happen to the microbiome in IBS.) Studies show that IBS patients have a different gut microbiota makeup, with decreased levels of beneficial bacteria and an increase in pro-inflammatory species [4,14]. Dysbiosis in IBS patients causes changes in microbial metabolites that impact the mucosal and systemic levels [11]. Visceral hypersensitivity and increased inflammatory cytokine production are frequent in PI-IBS [11]. Distinct microbial patterns have also been noted across IBS subtypes. In particular, one study discovered that fecal *Lactobacillus* and *Bifidobacterium* were correlated with IL-10 in IBS-C patients, while Gram-positive and Gram-negative bacteria are correlated with C-X-C motif chemokine ligand 11 in IBS-D patients [11]. The altered microbiota of some IBS patients may be associated with clinical severity and psychosocial factors; changes in brain regions related to emotional responses are correlated with changes in the microbiota, such as the prevalence of *Prevotella* over *Bacteroides* [11]. This microbiota is frequently unstable, influenced by environmental factors such as nutrition and antibiotic use, confounding comprehension of its function in the illness [5,14].

According to research, certain bacterial families, such as *Bifidobacteria* and *Faecalibacterium*, represented lower numbers than in healthy subjects, whereas *Lactobacilli* and *Bacteroides* were found to be increased [15]. IBS symptoms include decreased microbial diversity, increased pro-inflammatory species such as *Bacteroides*, and a decline in the number of anti-inflammatory species such as the butyrate-producing bacteria *Faecalibacterium prausnitzii* [16]. Subtype-specific differences in transcriptomics and metabolomics show various microbiota-related processes that underpin IBS symptoms. Additionally, one study showed an increase in *Clostridium* [17] and higher levels of *Streptococcus* and *Gardnerella vaginalis* [18], all of which are linked to IBS symptoms. One study indicated decreasing levels of *Lactobacilli* in IBS-D patients [19], while another mentioned a trend of decreased beneficial species such as *Lactobacilli* and *Bifidobacteria* in its findings, highlighting discrepancy [9]. Notably, IBS frequently results in a reduction in butyrate-producing bacteria and an increase in pro-inflammatory *Enterobacteriaceae* [8].

Through the fermentation of polysaccharides, *Methanobrevibacter* species like *Methanobrevibacter smithii* and *M. stadtmanae* play important roles in the gut microbiota by creating hydrogen (H₂) and methane (CH₄), which can affect gut permeability and bowel motility [2]. Furthermore, metabolite-sensing G-protein-coupled receptors (GPR43, GPR41, and GPR109A) interact with butyrate and other SCFAs generated by colonic bacteria, such as strains of *Bifidobacterium* and *Lactobacillus*, to control inflammatory responses and support gut homeostasis [2]. Because IBS is characterized by inflammation and immune system activation, these pathways are especially pertinent in this condition [2,11].

Methane-producing bacteria are more abundant in IBS-C than in IBS-D, with an overall decrease in butyrate-producing bacteria [9]. IBS patients had a higher *Firmicutes*-to-*Bacteroidetes* ratio and higher levels of certain *Streptococci* and *Ruminococcus* species than healthy individuals [9]. *Ruminococcaceae* levels have been found significantly reduced in IBS-D patients, as has bile acid metabolism, which is linked to symptoms such as diarrhea and visceral hypersensitivity [20].

Microbes and their constituents can enter the mucosa due to increased permeability caused by the intestinal epithelial barrier being disrupted in IBS [2,8,11]. The immune system is triggered by this exposure, which results in aberrant inflammatory reactions that exacerbate IBS symptoms [3,8,11]. This problem has been made worse by the discovery that IBS patients have abnormalities in tight junction proteins, which are essential for preserving barrier function [8,11]. IBS is characterized by immune activation in the intestinal mucosa, wherein pro-inflammatory cytokines are produced in greater quantities in response to either direct microbial stimulation or indirect activation via microbial antigens [8,11]. Visceral hypersensitivity and bowel pain are linked to this elevated immune response, which is biased toward pro-inflammatory Th1 and Th17 pathways [2,11]. Moreover, cross-reactive immune responses may be triggered by molecular mimicry between host proteins and pathogen antigens. For instance, host proteins like vinculin may be mistakenly targeted by antibodies produced against bacterial toxins, impairing intestinal neuronal activity [11]. Furthermore, by blocking histone deacetylases, microbial metabolites like butyrate can cause epigenetic modifications, modifying gene expression and adding to the molecular alterations observed in the gut and brain systems of individuals with IBS [11].

The gut–brain axis plays a part in the pathophysiology of IBS, as evidenced by elevated levels of pro-inflammatory cytokines such as IL-6, TNF-α, and IL-1β, which are associated with anxiety and depression [2]. Furthermore, intestinal permeability and somatic hypersensitivity are made worse by genetic and epigenetic variables, such as dysregulated microRNA production and changes in the serotonin receptor gene, which result in symptoms of the disease and a lower quality of life for IBS patients [2].

Despite proven abnormalities in gut microbiota in IBS patients, a particular microbial signature that distinguishes these individuals has yet to be found, with no clear signature identified for IBS subgroups [15,21]. Small intestine bacterial overgrowth (SIBO) is significantly more common in IBS patients [22], particularly in IBS-D [23]. However, its role is debatable due to diagnostic limitations [21].

The interplay of gut microbiota and bile acids (BAs) is important in IBS pathogenesis. Certain BAs, particularly CDCA and DCA, can cause cellular damage and compromise tight junction integrity, resulting in increased intestinal permeability that contributes to IBS symptoms [17]. According to research, impaired intestinal barrier integrity and increased immune activation may contribute to IBS symptoms, especially in instances triggered by past infections [11]. PI-IBS is a significant risk factor that can develop following a variety of gastrointestinal infections, with meta-analyses revealing a fourfold increase in IBS risk after infection [21]. PI-IBS can cause long-term symptoms due to dysbiosis and inflammation [23].

The gut microbiota has a major impact on intestinal barrier integrity and immune system modulation, two important aspects of IBS pathogenesis. In IBS patients, mast cells are more prevalent close to enteric nerve fibers [8,10]. These cells release mediators including serotonin and histamine, which cause cytokine imbalance and lymphocyte activation, changing pain thresholds and escalating visceral hypersensitivity [8,10]. By releasing tryptase, mast cell degranulation and eosinophil activation further weaken tight junction proteins, which increases intestinal permeability [8,10]. Certain microorganisms help to regulate these processes: tryptophan is converted by *Lactobacilli* species into indole-3-aldehyde, which activates the aryl hydrocarbon receptor (AHR), which controls intraepithelial lymphocyte populations and stimulates the production of the anti-inflammatory IL-22 [8].

Mucus layer composition and thickness are influenced by *Ruminococcus* species, *Bacteroides thetaiotaomicron*, and *Faecalibacterium prausnitzii* [8,18]. One of the most prevalent bacterial species in the gut is *Faecalibacterium prausnitzii*. Through the activation of regulatory T cells, the promotion of IL-10 secretion, and the inhibition of IL-8 synthesis, it demonstrates anti-inflammatory properties [8]. *Firmicutes* create SCFAs, which improve epithelial integrity by upregulating the expression of tight junction proteins such as occludin and claudins [8,17]. E-cadherin production is stimulated by polyamines produced by genera such as *Lactobacillus* and *Clostridium*, which strengthen barrier function [5,8]. *Lactobacillus rhamnosus*, *Bifidobacterium breve*, and other probiotic strains control pro- and anti-inflammatory cytokines, preserving the integrity of the intestinal barrier and reducing the symptoms of IBS [5,8].

Intestinal barrier dysfunction is common in IBS, particularly in IBS-D, and correlates with increased permeability, which contributes to low-grade inflammation and symptom exacerbation [24]. Tight junction protein expression changes and enhanced mast cell activation have been seen inside the intestinal mucosa [24]. Notably, SCFAs play an important function in increasing tight junction protein expression [8]. Dysbiosis can lead to the generation of proteases that weaken the intestinal barrier, emphasizing the intricate relationship between microbiota composition and gut integrity [24]. Furthermore, a reduction in the number of butyrate-producing bacteria may compromise intestinal barrier function [2].

Recent research found that higher serum levels of D-lactate and diamine oxidase are related to increased IBS severity [25], implying a possible relationship between microbiota composition and symptom intensity. Certain bacterial families, both beneficial and harmful, have been linked to IBS severity, supporting the concept of a microbiota-based biomarker for the illness. However, significant studies have found that IBS patients have an excess of harmful bacteria, such as *Enterobacteriaceae*, and fewer good bacteria, such as *Bifidobacterium* and *Lactobacillus* [25]. Dietary therapies, such as gluten-free or low fermentable oligosaccharides, disaccharides, monosaccharides, and polyols (FODMAP) diets, have not consistently improved dysbiosis indices in IBS patients [25], highlighting the complexities of the microbiome’s participation in the condition.

Research has also shown that psychological problems such as anxiety and depression are common among IBS patients, with a substantial relationship between symptom severity and psychological distress [2,13,26].

Food hypersensitivity adds to dysbiosis, complicating treatment options and highlighting the importance of personalized dietary approaches to properly control symptoms [27]. The gluten-free diet (GFD) has become popular among IBS patients, with some research suggesting symptom relief. However, this relief may be due to a reduction in fructans, which are FODMAPs rather than gluten itself, confounding dietary assessments [28].

In simple terms, changes in gut microbiota composition and functionality are critical to the pathophysiology of IBS, demanding additional research to understand these complex interactions. The link between gut microbiota composition and IBS symptoms is variable across the literature, underscoring the need for additional study to determine causality [15].

## 4. The Complex Relationship Between SIBO and IBS

Small intestinal bacterial overgrowth (SIBO), formerly known as “blind loop syndrome”, is associated with maldigestion and malabsorption caused by excessive bacterial growth in the small intestine. The symptoms are diarrhea, steatorrhea, and megaloblastic anemia. While jejunal aspirate cultures were formerly the diagnostic gold standard, breath tests like lactulose hydrogen (LHBT) and glucose hydrogen (GHBT) are now widely utilized despite concerns about specificity and false positives [29]. Studies indicate a high incidence of SIBO in IBS patients, while some report symptom relief following antibiotics; however, diagnostic inconsistencies confuse findings [29]. Emerging data have revealed a possible relationship between IBS and SIBO. Gastric achlorhydria, motility disorders, and small bowel stasis are all risk factors for SIBO [30]. While some studies suggest a high SIBO rate in IBS patients and relief of symptoms with antibiotics such as rifaximin, inconsistent evidence and the lack of defined diagnostic criteria confuse the SIBO-IBS link [30]. A case–control study found that 84% of IBS patients tested positive for LHBT, and neomycin therapy alleviated symptoms in these situations [30]. Similarly, the antibiotic rifaximin has shown minor efficacy in IBS-D and is approved by the FDA for this type of condition; however, its advantages do not directly indicate SIBO involvement. Systematic reviews have revealed uneven SIBO prevalence in IBS, with confounding factors such as proton pump inhibitor use aggravating the relationship [30]. Studies have found no obvious symptom differences between SIBO-positive and SIBO-negative people with IBS [30]. Current research does not clearly show SIBO as a causal factor in IBS, emphasizing the need for molecular techniques to investigate the intricate connection between gut microbiota and IBS pathology.

## 5. Biofilms and IBS

IBS, inflammatory bowel disease (IBD), and colorectal cancer (CRC) are among the gastrointestinal disorders that are influenced by biofilms, which are essential for preserving gut homeostasis. Comprising intricate microbial colonies shielded by a matrix, biofilms display traits including virulence and resistance to antibiotics that help prolong the course of disease [31]. To maintain host–microbiota equilibrium, these biofilms interact with the mucosal microbiota. Polymicrobial and trans-kingdom interactions (encompassing viruses, Prokarya, Eukarya, and Archaea) are key for host–microbiota balance [31]. Disruptions in the integrity of biofilms, frequently brought on by compromised mucus or excessive use of antibiotics, can result in dysbiosis and illness; resistance is increased by biofilm-associated bacterial dispersion and gene transfer, and the makeup of the gut’s mucosal microbiota differs from that of the fecal microbiota [31]. Understanding biofilm dynamics is crucial for therapeutic treatments, as biofilm development is especially common in high-density microbial regions like the colon [31].

Biofilms contribute to altered microbiomes and decreased bacterial diversity, which increase the density of bacteria in the gut mucosa and worsen disease pathophysiology [32]. Commensal biofilms can also be beneficial in preventing infections through competitive exclusion even if biofilms in pathogenic conditions like *H. pylori* directly contribute to the development and recurrence of the disease [32]. Moreover, IBS responds differently to therapies that target the gut microbiota, such as FMT, antibiotics, and dietary modifications. Rifaximin, for example, provides short-term symptom alleviation, especially for bloating [33]. However, the impact of these treatments on biofilm-related mechanisms remains poorly understood, and the inconsistent results from FMT trials suggest that biofilms are not fully addressed in current therapies [33]. Future research should focus on biofilm-targeted strategies to better understand and treat GI disorders like IBS.

## 6. Gut–Brain Axis and IBS Symptoms

The gut microbiota communicates bidirectionally with the autonomic nervous system (ANS), modulating gut motility and secretions through metabolites like serotonin, histamine, and GABA [22,34]. It affects both the central and enteric neural systems, impacting gastrointestinal function and emotional regulation. The microbiota–gut–brain (MGB) axis concept illustrates how these systems interact [3]. Neuroimaging studies utilizing MRI revealed structural and functional abnormalities in the brain associated with IBS, demonstrating that specific microbial signatures correspond to alterations in brain structure and activity [24]. Current research on the MGB axis has produced conflicting results owing to a lack of causal evidence tying changes in the gut microbiota to brain function. Most research have focused on preclinical animal models, indicating immune system interactions, metabolites, and neurotransmitter signaling as important communication pathways [3]. *Eubacterium*, *Bacteroides*, and *Clostridium* (clusters IV, XI, XIII, and XIVa) are key producers of secondary bile acids and SCFAs, which stimulate serotonin synthesis in colonic enterochromaffin cells and regulate gastrointestinal motility [2,8]. Serum serotonin levels differ between IBS subtypes, being lower in IBS-C and higher in IBS-D [3,5]. The serotonin system, particularly 5-HT3 and 5-HT4 receptors, plays a crucial role in gastrointestinal motility and sensory functions [6,8]. 5-HT4 receptors stimulate acetylcholine release, accelerating the peristaltic reflex, whereas 5-HT3 receptors mediate smooth muscle contraction and gut–brain communication [5,8]. Therapeutic interventions targeting these receptors have shown promise; 5-HT3 receptor antagonists alleviate abdominal pain and IBS-D symptoms, while 5-HT4 receptor agonists improve stool frequency, consistency, and abdominal discomfort in IBS-C [3,9]. However, adverse effects, including cardiovascular risks, have led to the withdrawal or restricted use of some medications, driving the development of safer alternatives [6]. Bacterial metabolites such as SCFAs impact neuropeptide production, which controls gastrointestinal motility and sensitivity [8].

Maternal factors, including as food and stress, have a significant impact on the baby microbiome, which affects brain and enteric nervous system development. The early microbiome makeup is essential for long-term gut–brain connection [3]. Research suggests that the timing of microbiota recolonization in germ-free rodents is critical for recovering key brain functions, implying a sensitive period for microbial influence [3]. While human research is scarce, preliminary results point to a link between microbiome composition and cognitive development, particularly in early childhood [3].

The human microbiota has been intensively investigated, with a revised human-to-microbiota cell ratio of 1.3:1, emphasizing microbial cells’ considerable genetic contribution [34]. Notably, more than 99% of the genes in the human body are microbial, implying a co-evolutionary relationship that could influence immune responses and epigenetics [34]. The gut microbiota can be therapeutically modified by diet and lifestyle, opening new avenues for treating chronic diseases, notably DGBI.

Communication between the gut and brain occurs via a variety of channels, including neurological, immunological, and metabolic mechanisms, with the vagus nerve playing an important role [13]. The vagus nerve has been identified as the principal pathway for gut microbiota impacts on the central nervous system (CNS), with various bacteria producing neurotransmitters that can influence behavior and brain function [34]. For example, several strains of *Bifidobacteria* have been demonstrated to increase tryptophan levels, a precursor to serotonin [34].

**Figure 2:**
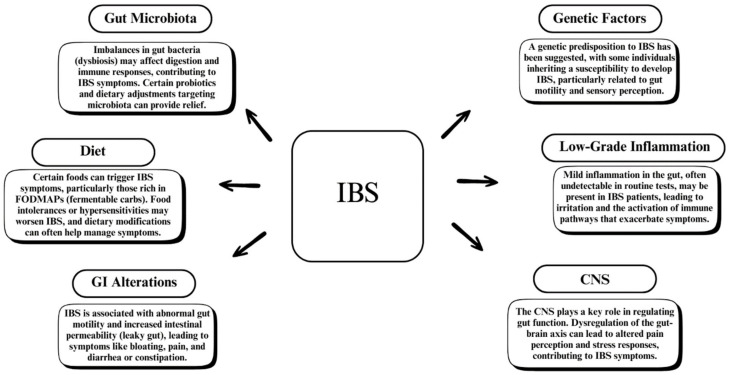
An overview of suggested factors involved in IBS.

The gut microbiota influences anxiety and stress-related behaviors, with significant differences in microbiome composition found in anorexia nervosa patients compared to healthy controls. (Figure 2 shows an overview of other factors that could be involved in IBS pathogenesis.) Evidence suggests a link between gut microbiota and neurodegenerative illnesses, with certain bacterial species influencing brain health and function [35]. Furthermore, neuropsychological symptoms such as brain fog have been linked to increased SIBO rates, implying a possible gut–brain connection [36].

## 7. Microbiome Targeted Treatment in IBS

Probiotics have been demonstrated to boost immunological function, reduce inflammation, and promote gut health in IBS patients by increasing the number of helpful bacteria and promoting the production of SCFAs [20]. Specifically, strains such as *Bifidobacterium* and *Lactobacillus* have shown promise in alleviating IBS symptoms, although their effectiveness varies depending on the strain and patient characteristics [1,37,38]. Probiotics are thought to lower gut inflammation, enhance gut health, and regulate immune responses by modulating pro-inflammatory and anti-inflammatory cytokines [10,39]. Probiotics increase short-chain fatty acids, promote *Lactobacillus* and *Bifidobacterium* colonization, and alleviate colonic hypersensitivity by upregulating μ-opioid and cannabinoid receptor expression. They also enhance gut barrier function, inhibit pathogenic bacteria, produce neurotransmitters, and regulate IL-10/IL-12 levels while decreasing pro-inflammatory cytokines [15,20]. Moreover, certain probiotics may alter gut pain receptors and affect the immune system and brain, contributing to symptom relief, mood improvement, and anxiety reduction [7,39].

While probiotics hold potential, research remains inconsistent. For example, a meta-analysis of 54 randomized controlled trials (RCTs) found that probiotics, particularly *Lactobacillus* and *Bifidobacterium* strains, significantly improved IBS symptoms, especially abdominal pain, but effects varied across strains and formulations [38,40]. In placebo-controlled studies, *B. infantis* significantly improved abdominal pain/discomfort in IBS patients after at least 4 weeks of treatment; *B. lactis* reduced abdominal distension, transit times, pain/discomfort, and global IBS symptoms in female IBS-C patients; *B. animalis* improved bloating within 3 weeks and stool frequency within 6 weeks in IBS-C patients; and *B. bifidum* improved pain, discomfort, bloating, urgency, and quality of life after 4 weeks of treatment [37]. Also, placebo-controlled trials demonstrated that *Lactobacillus* probiotics (*L. plantarum*, *L. rhamnosus*, *L. casei*, and *L. reuteri*) alleviate IBS symptoms, but the outcomes are less consistent compared to *Bifidobacterium* [37]. Another meta-analysis indicated that some probiotics may be useful, and combining probiotics with certain strains relieved symptoms, gastrointestinal discomfort, and bloating [41]. Recent studies have suggested that probiotic combinations are more beneficial than single-strain options, with *Escherichia* and *Streptococcus* combinations resulting in reduced abdominal pain and bloating [7,38]. Nonetheless, the efficacy of probiotics appears to depend on the strain, dosage, and duration of treatment, with some patients experiencing only temporary symptom relief [15,37].

Interestingly, probiotics’ ability to promote gut health may extend beyond symptom relief. The composite IBS symptom score decreased significantly in the probiotic group compared to placebo [42]. Probiotic treatment resulted in a 37% reduction in IBS score versus 9% in placebo [42]. They can increase levels of beneficial bacteria like *Bifidobacterium* and *Lactobacillus*, producing SCFAs that are vital for intestinal function [10]. However, the overall effectiveness of probiotics remains inconclusive, with studies highlighting the need for more research to identify the most effective strains and dosages for IBS management [19,43]. Synbiotics, which combine probiotics and prebiotics, have shown promise by providing additional benefits through synergistic effects, improving stool frequency, and reducing bloating [2].

## 8. Prebiotics in IBS

Prebiotics, non-digestible fibers that promote the growth of beneficial gut bacteria, have shown potential in treating IBS symptoms, particularly bloating and gas [20]. Prebiotic fermentation produces SCFAs, which possess anti-inflammatory properties that may help alleviate IBS symptoms [44]. Clinical trials have indicated that low to moderate doses of prebiotics can relieve symptoms, although large doses may exacerbate bloating and other gastrointestinal discomforts [44,45].

Similar to probiotics, the efficacy of prebiotics in IBS treatment is not uniform across studies. For instance, supplementation with fructooligosaccharides (FOS) and galactooligosaccharides (GOS) promotes the growth of beneficial bacteria, but symptom relief has been inconsistent [36]. Some patients report improvements in bloating and gas with low doses of trans-GOS, but higher doses can increase symptoms due to fermentation [36]. This highlights the importance of individualized approaches when using prebiotics to manage IBS.

## 9. Synbiotics and Postbiotics

Synbiotics, which combine prebiotics and probiotics, aim to enhance the efficacy of both by improving probiotic survival in the gastrointestinal system [44]. This synergistic approach shows potential in optimizing IBS treatment, with some studies indicating improvements in bowel movement frequency and reductions in bloating [2,45]. However, more research is needed to understand the long-term effects and optimal formulations of synbiotics for IBS patients [5].

In addition to synbiotics, postbiotics, i.e., metabolites produced by probiotics, are emerging as potential therapies for IBS. Although the research on postbiotics is still in its infancy, early findings suggest that they may help reduce inflammation and improve symptoms, especially in diarrhea-predominant IBS [11]. This innovative approach warrants further investigation to determine its effectiveness in clinical settings.

## 10. Fecal Microbiota Transplantation (FMT) in IBS

**Figure 3:**
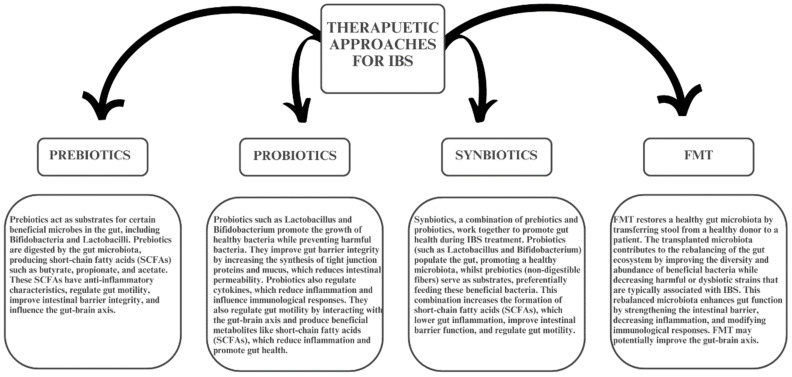
Summary of the mechanisms of action of different therapeutic approaches for IBS.

FMT has gained attention as a potential therapy for IBS by restoring gut microbiota composition. (Figure 3 summarizes the mechanism of action of different therapeutic approaches for IBS.) Several studies have shown that FMT can result in significant symptom improvements, particularly in patients with severe IBS or high levels of gut dysbiosis [7,46,47]. However, the outcomes of FMT are inconsistent. Some trials report substantial reductions in IBS symptoms, while others show minimal or no effects compared to placebo [9,36,48]. These discrepancies may be influenced by factors such as donor selection, delivery method, and individual patient characteristics [25,48].

The mode of FMT administration also appears to play a crucial role in determining its efficacy. For example, older FMT techniques, such as enema or colonoscopy, have been found to be more effective than newer methods like capsule delivery [36,47]. Multiple-donor FMT has shown promise, but its overall effectiveness in treating IBS remains inconclusive [48]. Moreover, the long-term consequences of FMT on gut microbiota and IBS symptom relief are still unknown, necessitating further research to confirm its safety and efficacy [40,43,46].

Despite its potential, FMT’s clinical application in IBS management is not without challenges. For instance, male patients have shown reduced response rates to FMT, while patients with severe IBS have reported better outcomes [25]. Additionally, certain bacterial profiles in donors may predict treatment efficacy, highlighting the need for personalized approaches to FMT therapy [25,49].

## 11. Dietary Interventions for IBS

Dietary interventions, particularly the low-FODMAP diet, are among the most effective first-line treatments for IBS. FODMAPs, or fermentable oligosaccharides, disaccharides, monosaccharides, and polyols, are poorly absorbed carbohydrates that can cause bloating, gas, and discomfort in IBS patients. The low-FODMAP diet is typically implemented in three phases: exclusion, gradual reintroduction, and long-term personalization to meet individual needs [28].

Studies have shown that the low-FODMAP diet can significantly reduce IBS symptoms, with symptom reduction rates ranging from 50% to 76% during the initial elimination phase [15,28]. Long-term adherence to the diet, particularly with the guidance of a dietitian, can provide sustained symptom relief, especially from fructans [15,28]. Dietary factors play an important role in IBS patients, with meal-related symptom aggravation frequently observed, and specific foods such as high-fat meals and poorly digested carbohydrates trigger symptoms through fermentation and modified colonic responses [50]. However, the low-FODMAP diet is not without risks. Long-term use may result in nutrient deficiencies, alterations in gut microbial diversity, and reduced levels of beneficial bacteria such as *Bifidobacterium* [26,28,49].

The efficacy of the low-FODMAP diet varies depending on IBS subtype. For instance, while it significantly alleviates symptoms in diarrhea-predominant IBS patients, it may be less effective in those with constipation-predominant IBS [29,32]. Furthermore, research comparing the low-FODMAP diet to traditional dietary guidance has yielded conflicting results, suggesting that other dietary approaches may be equally beneficial for some patients [25].

In contrast to the low-FODMAP diet, the NICE diet has been proposed as a better long-term solution for IBS patients, benefiting 46% to 54% of patients while avoiding the nutritional risk factors associated with FODMAP restriction [51]. The NICE diet focuses on balanced nutrition, avoiding the stringent restrictions of the low-FODMAP diet, which can lead to calorie restriction and nutritional deficits [51]. Despite the challenges of maintaining the low-FODMAP diet long-term, it remains one of the most effective dietary therapies for IBS symptom management [15].

## 12. Gluten-Free Diet (GFD) and IBS

Wheat grains contain a variety of components, including proteins such as gluten, which is composed of glutenin and gliadin. Barley, rye, and oats all contain gluten-like proteins known as hordein, secalin, and avenins. Wheat also contains albumins, such as amylase-trypsin inhibitors (ATIs), and starch, which includes fructans, an oligosaccharide classed among the FODMAPs. As a result, people who consume a wheat-based diet are exposed to gluten proteins, ATIs, and FODMAPs, all of which may contribute to gastrointestinal discomfort in IBS patients [27]. This complication makes it difficult to pinpoint the exact components responsible for symptom relief when wheat is removed from the diet [27].

Celiac disease is caused by a particular immune response to gluten, a wheat protein breakdown product that binds to the HLA-DQ2 and HLA-DQ8 receptors on antigen-presenting cells. This interaction results in a mucosal inflammatory response that includes lymphocyte infiltration, crypt enlargement, villous atrophy, and accelerated epithelial cell turnover. IBS symptoms have been connected to an immunological response to gluten, which is comparable to celiac disease; however, the exact mechanism is yet unknown [26]. Prior to starting a gluten-free diet, celiac patients also exhibit key abnormalities seen in IBS, such as increased gut permeability, higher mucosal mast cells, and decreased serotonin transporter expression [26].

Some IBS patients report symptom alleviation from GFD, although the underlying mechanism remains uncertain. Patients with IBS are 3.5 times more likely than controls to report gluten intolerance [26]. Research suggests that reducing fructans rather than gluten may be responsible for the observed symptom relief in non-celiac gluten-sensitivity (NCGS) patients [28]. Despite this, many patients continue to adhere to the GFD long-term, even though it may lead to nutritional deficiencies and potential heavy metal accumulation [28].

While the GFD may provide symptom relief for some IBS patients, its long-term safety and effectiveness are still debated. As with other dietary interventions, individualized approaches are essential to minimize risks and ensure proper nutrition [28,49].

## 13. Personalized Approaches in IBS Management

The emerging trend in IBS management is the shift towards personalized treatment approaches that consider individual differences in gut microbiota and dietary responses [21,52]. For instance, the gut microbiome may predict a patient’s response to the low-FODMAP diet, with higher dysbiosis scores indicating a poor response [9,28]. Similarly, pre-treatment gut flora diversity may serve as a biomarker for predicting the efficacy of FMT in IBS patients [25,46].

Personalized nutrition programs that balance symptom relief with adequate nutrition are vital for long-term IBS management. These programs must be tailored to the patient’s unique microbiome composition, symptom profile, and dietary preferences [49]. As research progresses, the integration of microbial and dietary therapies holds promise for improving IBS management and patient outcomes [52].

## 14. Challenges and Future Research Directions in Microbiome-Targeted Treatments for IBS

The study of microbiome-targeted treatments for IBS has advanced significantly, yet substantial gaps remain in our understanding. These knowledge gaps impede the ability to develop personalized treatment strategies, which could address the diverse subtypes and symptom profiles of IBS. This section highlights key challenges in current research, focusing on the gaps in knowledge and potential avenues for personalized treatments.

## 15. Gaps in Current Knowledge

One of the major challenges in IBS research is the lack of long-term data on the effects and adverse events related to probiotic interventions. Most existing studies on probiotics for IBS treatment are short-term and do not adequately assess the variability in probiotic formulations or the heterogeneity in patient responses [53]. This is problematic because the effectiveness of probiotics can vary significantly based on the strain used, dosage, and treatment duration, which are not yet optimized for specific IBS subtypes [53]. Additionally, the safety profile of probiotics, particularly with long-term use, remains unclear. More extensive trials with longer follow-up periods are needed to monitor potential side effects and adverse events, which are crucial for refining probiotic therapeutic strategies [53].

Another limitation in the current research is the small sample sizes and short study durations, which restrict the ability to draw robust conclusions, particularly about the efficacy of probiotics across different IBS subtypes. Studies with larger populations and longer durations are essential to evaluate the long-term effectiveness of probiotics and to determine their impact on various IBS subtypes [54]. Furthermore, current trials often focus solely on probiotics, neglecting other potentially beneficial agents such as fiber and prebiotics. The inclusion of these components in future studies could provide a more comprehensive understanding of how various interventions impact IBS symptoms and gut health [54].

In addition to these methodological challenges, there are significant gaps in our understanding of the microbiome–brain interaction, particularly in the context of DGBI, including IBS. The current research on microbiome function and its role in IBS pathophysiology is hampered by inconsistent methodologies for assessing the microbiome, which leads to conflicting results [10]. To address this, large-scale longitudinal studies using multi-omics approaches are needed to investigate the interactions between the microbiome and the host in IBS patients. Future studies should focus on improving the timing and integration of psychological therapies into treatment plans in order to maximize their function alongside medical treatment [55]. Such studies could explain the role of gut–brain communication mechanisms and the potential for microbiome-targeted therapies, such as FMT and psychological interventions, in IBS management [13].

Geographical and population-specific differences also represent a significant gap in IBS research. There is a notable lack of studies focused on IBS patients from Asia, where the microbiota composition may differ due to genetic, dietary, and environmental factors [25]. This underrepresentation limits the generalizability of current findings. Future studies should explore how dietary interventions influence microbiota changes across different IBS subtypes and investigate the efficacy of probiotics and FMT in these populations [25]. Additionally, the role of beneficial bacteria in the pathogenesis of IBS requires further research to determine whether certain strains could be leveraged for targeted therapeutic interventions [25].

## 16. Potential for Personalized Treatments

Personalized treatments for IBS, which account for individual variations in microbiota composition and host factors, hold great promise but remain an underdeveloped area of research. Current findings often fail to meet clinical needs, highlighting the necessity for further exploration of the specific pathophysiological mechanisms underlying IBS [49]. The complexity of IBS pathophysiology, which can differ significantly between patients, calls for a deeper investigation into how personalized medicine can be integrated into IBS treatment strategies. (Table 1 displays the results of different meta-analyses.) For example, the efficacy of FMT varies across different IBS subtypes, and there is a need to examine how individual patient characteristics, such as microbiome composition, influence treatment outcomes [49]. Similarly, the response to probiotic therapy may differ based on individual genetic and microbial profiles, suggesting that treatments should be tailored to these factors [49].

One of the key challenges in developing personalized treatments is the lack of clarity regarding the optimal probiotic combinations for managing IBS symptoms. While probiotics are commonly used in IBS treatment, it is still unclear which strains, dosages, and combinations are most effective for specific patient subtypes and symptom profiles [40]. This highlights the need for more clinical trials focusing on the efficacy of particular probiotic strains and combinations tailored to different forms of IBS [40]. Such research could benefit from integrating genomic and transcriptomic analyses to better understand the molecular mechanisms of probiotics and their interaction with the host microbiome [40].

Geraniol, an essential oil constituent, has anti-inflammatory, antibacterial, and eubiotic effects on gut microbiota in IBS patients. In a randomized double-blind trial, geraniol reduced overall IBS symptoms and gut microbiota profiles, especially in IBS-M patients [25]. Clinoptilolite, a natural zeolite that has high absorptive ability, was found to reduce diarrhea frequency in a randomized trial of IBS-D patients. A new double-blind RCT investigated the efficacy of GTB1 in 27 IBS-D patients. After four weeks, the GTB1 group’s abdominal pain and bloating severity decreased significantly. GTB1 also enhanced the relative abundance of *Lactobacillus* while decreasing *Bacteroides* levels within one week of therapy [25]. Furthermore, randomized trials have suggested that fiber can assist IBS-C patients, with viscous fiber (psyllium) improving stool consistency and frequency by increasing water content [26].

Furthermore, the use of FMT as a treatment for IBS remains in its early stages, with few studies investigating its efficacy across IBS subtypes. Future research should focus on identifying the optimal donor selection criteria, treatment regimens, and bacterial strains for effective FMT therapy in IBS [40]. A more personalized approach to FMT, which considers individual variations in gut microbiota, could significantly improve treatment outcomes for IBS patients [40].

The incorporation of personalized medicine into IBS treatment also requires advancements in understanding how microbiome alterations can affect the broader gut–brain axis. There is still much to learn about how the gut microbiota interacts with the central nervous system and how these interactions influence IBS symptoms. Psychological therapies, including cognitive behavioral therapy (CBT) provided over the phone, have shown long-term efficacy in treating IBS symptoms [55]. Investigating the mechanisms behind these microbiome–brain interactions, particularly in the context of psychological therapies, could open up new avenues for personalized treatment options [13].

## 17. Conclusions

IBS is a multifactorial disorder influenced by a combination of genetic predispositions, gut microbiota alterations, and psychosocial factors. The growing body of evidence supporting the role of the gut microbiota, particularly its involvement in the gut–brain axis, provides valuable insights into the pathogenesis and management of IBS. While microbiota-targeted therapies such as probiotics, synbiotics, and FMT show promise, the variability in patient responses underscores the need for more personalized treatment approaches. Future research should focus on identifying specific microbial signatures that can serve as biomarkers for IBS and exploring the long-term efficacy and safety of these interventions. A more nuanced understanding of the microbiota’s role in different IBS subtypes will be crucial for optimizing individualized therapeutic strategies and improving patient outcomes.

## Figures and Tables

**Table 1:** A summary of several meta-analyses evaluating the role of microbiota in IBS.

Reference	Methods	Results
Myneedu et al. 2019 [47]	Authors searched PubMed, Embase, Google Scholar, and abstract books from Digestive Disease Week and United European Gastroenterology Week (2010–2018) for studies on IBS, and they retrieved single-arm and RCTs on FMT for IBS, where the diagnosis was confirmed by a physician or based on ROME I-IV criteria.	Following the SATs, almost 60% of IBS patients reported considerable symptom relief. However, the RCTs revealed varied findings. Some studies indicated improvements in symptoms, while others found no significant difference between the FMT and control groups.
Zhang et al. 2022 [52]	Authors searched for RCTs on the efficacy of probiotics in treating IBS until August 25, 2021. The primary focus was on the rate of symptom reduction as well as changes in overall symptoms. Meta-regression was used to determine whether the length and dose of probiotic treatment had an impact on effectiveness.	*B. coagulans* was found to be the most effective probiotic species for relieving IBS symptoms. *L. plantarum* was shown to confer the highest quality of life (QOL). Meta-regression revealed that probiotic dose had no significant impact on outcomes, whereas treatment time did. Further subgroup analysis found that *B. coagulans* given for 8 weeks was the most effective in relieving these symptoms, outperforming probiotic combinations in the research.
Shang et al. 2022 [39]	Authors conducted a comprehensive search of PubMed, Embase, the Cochrane Library, Web of Science, and China Biology Medicine (CBM). Intervention parameters included probiotic strains, dose, duration, form, and placebo use as well as outcome measures such as symptom reports and scale use.	Three RCTs with 71 patients found that probiotics significantly improved stool consistency compared to placebo. An 8-week therapy period was effective, while 12 weeks provided no benefit. Two RCTs with 74 patients found that probiotics significantly improved fecal *Bifidobacterium* and *Lactobacillus* levels after four weeks. There was no effect after 8 weeks.
Xie et al. 2023 [38]	Reviewers independently gathered crucial information from eligible trials, such as RCT details, participant characteristics, and results. Intention-to-treat analyses were conducted. Transitivity was established by comparing major clinical variables between studies. The network’s consistency was verified by node splitting and loop-specific analysis.	The most effective probiotics were *L. acidophilus* (efficacy level A). Other strains, such as *B. bifidum* and *C. butyricum*, provided considerable benefits (efficacy level B). The multistrain group demonstrated the greatest improvement in quality of life. *C. butyricum* also showed significant improvements over placebo. *B. coagulans MTCC 5856* and *S. cerevisiae CNCM I-3856* were found to be most effective at improving stool consistency in IBS-D patients. There were no significant differences between probiotics and placebo for IBS-C in this network analysis.
Jamshidi et al. 2023 [48]	A complete search of the PubMed/Medline and Embase databases was performed to include all relevant publications up to 14 June 2023. To reduce heterogeneity, subgroup analyses were conducted based on FMT preparation, frequency of administration, and route of administration.	Single dosage of FMT administered via colonoscopy significantly decreased patient complaints.Using frozen FMT as an oral capsule significantly increased symptoms compared to the non-FMT placebo.Patients undergoing multiple-donor FMT showed considerable improvement compared to autologous. However, compared to the non-FMT placebo, it had a negative effect on IBS symptoms.
Wu et al. 2024 [40]	Databases were searched, including Medline, Embase and Embase Classic, Cochrane Central Register of Controlled Trials, and Web of Science. Authors also searched for unpublished trial data on ClinicalTrials.gov. Standardized mean differences (SMDs) were utilized to compare the effect sizes of the experimental and placebo groups. The study looked at the effects of several probiotic strains by dividing interventions into subgroups based on the strain.	Probiotics had the greatest treatment benefit over placebo. FMT also showed considerable improvement. Prebiotics did not differ significantly from placebo. Synbiotics also did not reveal significant differences from placebo. Probiotics performed much better than prebiotics and synbiotics. FMT performed much better than prebiotics and synbiotics. Probiotics helped to improve abdominal pain and bloating when compared to placebo.
